# Evolutions in Microstructure and Mechanical Properties of Ultra-Thin Oligocrystalline Invar Alloy Strip During Cold Rolling

**DOI:** 10.3390/ma18092026

**Published:** 2025-04-29

**Authors:** Jianguo Yang, Yajin Xia, Qingke Zhang, Genbao Chen, Cheng Xu, Zhenlun Song, Jiqiang Chen

**Affiliations:** 1 School of Materials Science and Engineering, Jiangxi University of Science and Technology, Ganzhou 341000, China; yangjianguo@nimte.ac.cn (J.Y.); hermit_01@163.com (J.C.); 2 State Key Laboratory of Advanced Marine Materials, Ningbo Institute of Materials Technology and Engineering, Chinese Academy of Sciences, Ningbo 315201, China; xucheng@nimte.ac.cn (C.X.); songzhenlun@nimte.ac.cn (Z.S.); 3 Haiyan Zhongda Electronic Materials Co., Ltd., Jiaxing 314312, China; xyj21895@163.com; 4 Yongxing Special Materials Technology Co., Ltd., Huzhou 313005, China; yxteganghui_2016@126.com

**Keywords:** invar alloy, ultra-thin strip, cold rolling, plastic deformation, surface morphologies, tensile properties

## Abstract

The ultra-thin Invar alloy strips are widely used in the manufacture of the fine masks; cold rolling of such thin strips (<100 μm) poses significant difficulties, primarily due to the limited number of grains within the thickness range. Consequently, it is important to understand the grain structure and property evolutions of the ultra-thin Invar alloy strips during cold rolling. In this study, an annealed Invar36 alloy strip, 100 µm thick, was cold rolled to different thicknesses, and the surface deformation morphologies, cross-sectional grain structure, intracrystalline microstructure and tensile properties of these thin strips were characterized and analyzed. The results show that plastic deformation of the initial annealed equiaxed grains is not uniform, depending on the grain orientation, resulting in different slip bands morphologies, unevenness and increase in roughness. Meanwhile, the grain rotation and rolling texture develop with increasing cold rolling reduction. The dislocation density in the 60% cold-rolled strip is about decuple that of the original annealed strip, and high-density tangled dislocations are formed, making the tensile strength increase from 430 MPa to 738 MPa. Grain refining and proper intermediate annealing are proposed to optimize the thickness uniformity, evenness and surface roughness.

## 1. Introduction

The Invar alloys, primarily composed of the Fe and Ni elements, are renowned for their extremely low thermal expansion coefficient [1]. With the rapid advancement of micro-manufacturing trends, the Invar alloys have regained substantial attention [2], and have been applied in aerospace, precision instruments, mold manufacturing, optical components, electronic devices, etc. [3,4,5,6]. In recent years, the Fe36Ni Invar alloy strips are widely used in the manufacture of the fine evaporation masks for high-resolution organic light-emitting diode (OLED) display panels, for which both high strength, ultra-thin, low inner stress, high uniformity and superior strip shape are required [7,8]. With the rapid development of the OLED industry, there is an urgent demand for high-quality ultra-thin Invar alloy strips.

The ultra-thin high-strength Invar alloy strips, like the other thin alloy strips, are produced with precise cold rolling as the final forming method [9,10,11]; thus, the microstructure and property evolution of the thin Invar alloy strips during the precise cold rolling are critical [12]. However, the existing investigations on cold rolling of the Invar alloys predominantly focus on the medium-thick plates [13,14]. In the studies on precise rolling of the thin Invar alloy strips, the main research emphasis is on grain structure, phase and texture [12,15]. However, the precise rolling of very thin strips (<100 μm) is difficult because there are only a few grains (oligocrystalline) within the thickness range, and non-uniform deformation and crinkles are easier to occur during the rolling process [16,17]. Therefore, comprehensive understanding of plastic deformation and strength mechanisms of the oligocrystalline thin strips is the key point for precise rolling.

For the reasons above, in this study, an annealed Invar alloy strip, 100 µm thick, was cold rolled to different thicknesses, and the surface morphologies, roughness, cross-section grain structure, grain orientation maps, texture, phase, intracrystalline microstructure, tensile properties and fracture behaviors of these thin strips with different cold rolling reduction were comprehensively characterized. Based on that, the evolution in microstructure and mechanical properties of the ultra-thin Invar alloy strip during the cold rolling process are analyzed. It is hoped that this study can establish some foundation and practical guidelines for optimizing the rolling technology of the ultra-thin Invar alloy strips.

## 2. Materials and Methods

### 2.1. Preparation of Materials

The original strip used in this study is an annealed AISI/ASTM Invar36 alloy with a thickness of 100 µm, which was produced by the Haiyan Zhongda Electronic Materials Co., Ltd. (Jiaxing, China), and its composition, characterized by X-ray Fluorescence Spectrometer (XRF, Bruker S8 TIGER, Karlsruhe, Germany), is shown in Table 1. To prepare the cold rolling specimens, the original strip was first cut into specimens 15 cm × 10 cm in size, and then the lubricating oil, purchased from Yiwu Biqi Lubricant Co. (Yiwu, China), was evenly applied to the surfaces of both the roller and specimens. The rolling equipment employed is a laboratory rolling mill with the largest rolling width of 200 mm. After each pass, the roller was lifted and thoroughly cleaned, and fresh lubricant oil was reapplied to ensure complete separation and no adhesion. Two different rolling reductions were used at different stages: 10 µm for each pass when the strip was cold rolled from 100 μm to 70 μm, and 5 µm for each pass from 70 μm to 40 μm. After multiple passes of rolling, the specimens were gradually rolled to the target thicknesses of 90 µm, 80 µm, 70 µm, 60 µm, 50 µm and 40 µm, respectively, and there was no annealing during the whole rolling process.

### 2.2. Characterizations

After the cold rolling, the surface morphologies of all the specimens were cleaned and observed by Optical Microscope (OM, NMM 800RF, Ningbo, China), Scanning Electron Microscopy (SEM, Zeiss Sigma 300, Oberkochen, Germany) and the Laser Confocal Microscopy (LCM, Zeiss LSM700, Oberkochen, Germany). The phase and grain orientation of the specimens were characterized using X-ray diffraction (XRD, D8 DISCOVER, Karlsruhe, Germany). To reveal the cross-sectional grain structure and the surface relief, small pieces of the cold-rolled strips were vertically embedded in resin, mechanically ground, polished and corroded with a 10% FeCl_3_ solution for 80 s, and then observed using OM. To show the grain orientation and the dislocation density, the original specimen and the 60% cold-rolled specimen (40 µm) were sampled, and their surfaces were polished by ion beam polishing instrument (Leica, EM TIC 3X, Weztlar, Germany) and then characterized by Electron Back Scatter Diffraction (EBSD, Thermo Scientific Verios G4 UC, Waltham, MA, USA) equipped on the SEM. The obtained data were processed to obtain the grain map, grain size distribution and dislocation density. To show the intracrystalline microstructure, the original and 60% cold-rolled specimens were manually ground to a thickness of 40 μm, and then an ion thinning instrument (MODEL, Gatan691, Pleasanton, CA, USA) was used for thinning. The dislocation configurations of the specimens were observed by a Transmission Electron Microscope (TEM, ThemoFisher Talos F200x, Waltham, MA, USA).

### 2.3. Mechanical Properties Tests

The tensile tests were conducted using a universal tensile test machine (Zwick/Roell Z1.0, Shanghai, China). Five specimens were selected for each thickness to obtain the average strength. The strips were cut into specimens with the size of 10 cm × 1 cm along the rolling direction. The cross-beam speed of the tensile tests was set to be 0.1 mm·min^−1^. The fracture surfaces and side surfaces were observed by SEM to reveal the plastic deformation and fracture mechanisms. The microhardness of the strips was measured with the microhardness tester under a load of 50 g, and 10 indentations were tested to obtain the average value.

## 3. Results and Discussion

### 3.1. Evolution in Surface Morphology

**Figure 1:**
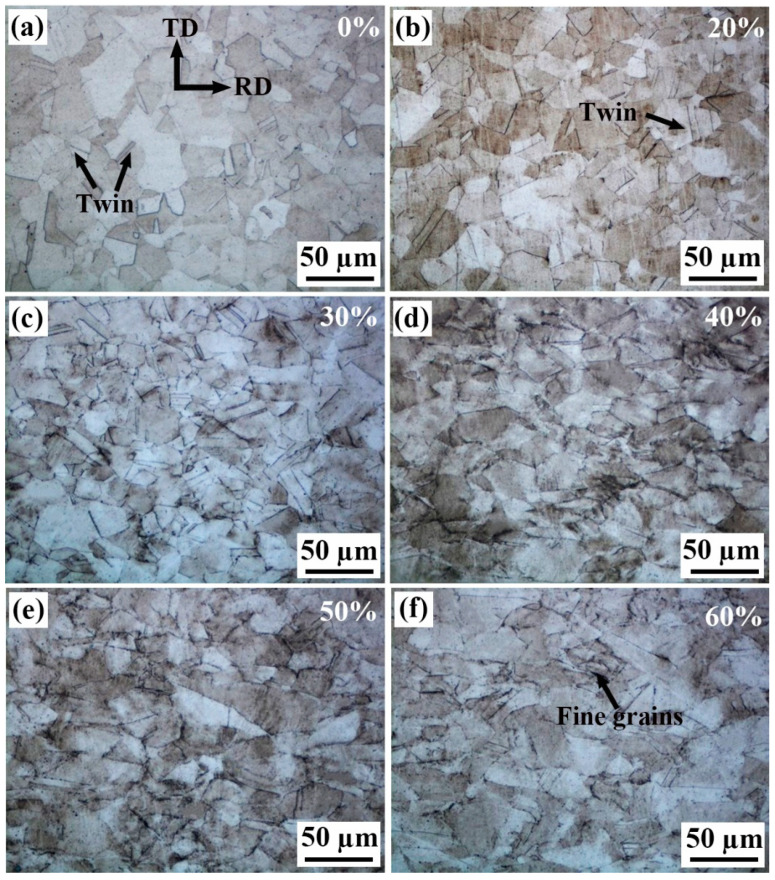
Surface appearance of the Invar alloy strips with different cold rolling reductions (%): (**a**) original strip, 100 μm (0%), (**b**) 80 μm (20%), (**c**) 70 μm (30%), (**d**) 60 μm (40%), (**e**) 50 μm (50%) and (**f**) 40 μm (60%).

Figure 1 shows the surface morphologies of the Invar alloy strips observed by OM, with the cold rolling reduction marked on the corner. It can be seen from the figure that for the original annealed strip, the grains are mostly equiaxed and the grain boundaries (GBs) are flat and clear, with the grain size ranges from about 20 µm to 40 µm, and there are some annealing twins, as shown in Figure 1a. After the cold rolling, the surface GBs become less clear, and the grains are elongated along the rolling direction (RD) with increasing cold rolling reduction, as shown in Figure 1b–d. Meanwhile, the GBs gradually become a little bit twisted, because there is inevitably some grain rotation during the cold rolling process, resulting in the rotation and twisting of the GBs. In addition, due to the high plastic deformation, a large number of dislocations will be generated; some of the dislocations pile up around the GBs, and thus the GBs become blurred. When the cold rolling reduction increases to 50~60%, it seems that some grains become crushed into fine grains, as shown in Figure 1e,f. Even inside the grains, the metallographic structure becomes less clear as the slip bands and some other deformations, which were further revealed by SEM, appear on the grain surfaces.

**Figure 2:**
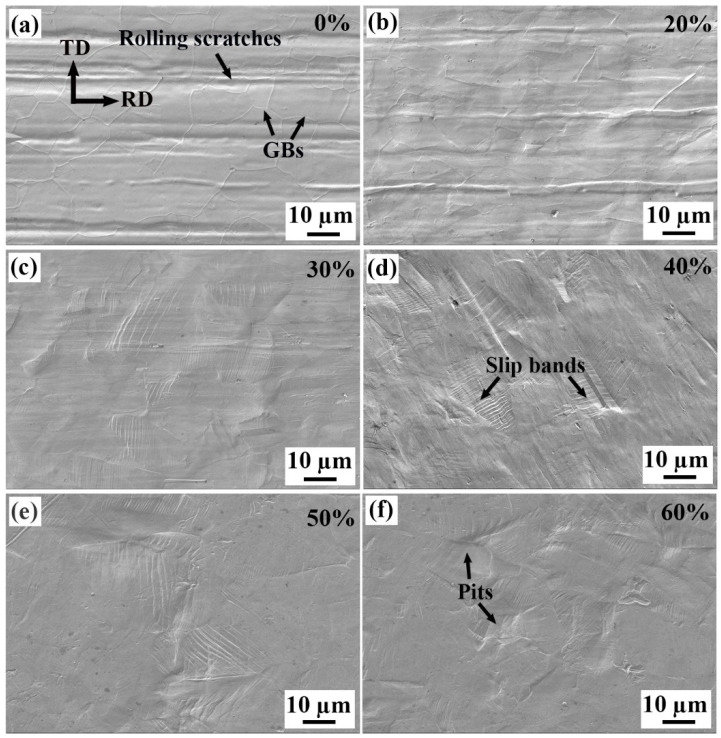
Surface deformation morphologies of the Invar alloy strips with different cold rolling reductions (%): (**a**) original strip, 100 μm (0%), (**b**) 80 μm (20%), (**c**) 70 μm (30%), (**d**) 60 μm (40%), (**e**) 50 μm (50%) and (**f**) 40 μm (60%).

The surface morphologies of the strips observed by SEM are shown in Figure 2. For the original strip, both the GBs and the previous rolling scratches are clear (see Figure 2a), indicating that the recrystallization and growth of the grains during the previous recrystallization annealing process do not affect the surface scratches. During the cold rolling, the surface scratches are subjected to large compressive stress and are gradually flattened, and the GBs become less clear, as shown in Figure 2b. With higher cold rolling reduction, parallel slip bands appear on the surface, and the original scratches and GBs disappear (see Figure 2c). Obviously, the distribution of the slip bands is not uniform, because the slip bands result from dislocation slip, while plastic deformation is not uniform within the different grains. Also, the non-uniform plastic deformation makes the surface of the strips uneven, which can be seen in Figure 2d–f, because the yield strength, elastic deformation and spring back of each grain are different. As a result, some “pits” appear on the surface of the strip, as marked in Figure 2f, which will significantly increase the surface roughness.

**Figure 3:**
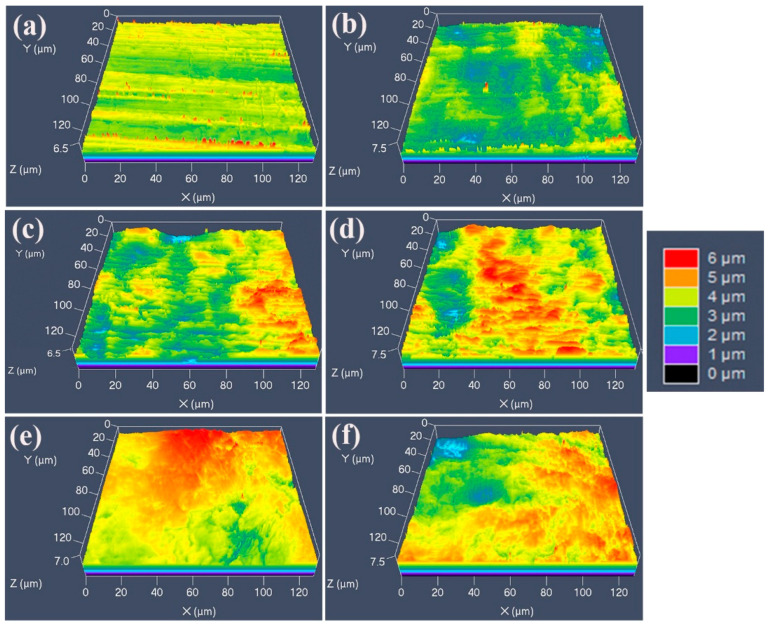
Three-dimensional surface morphologies of the Invar alloy strips with different cold rolling reductions (%): (**a**) original strip, 100 μm (0%), (**b**) 80 μm (20%), (**c**) 70 μm (30%), (**d**) 60 μm (40%), (**e**) 50 μm (50%) and (**f**) 40 μm (60%).

**Figure 4:**
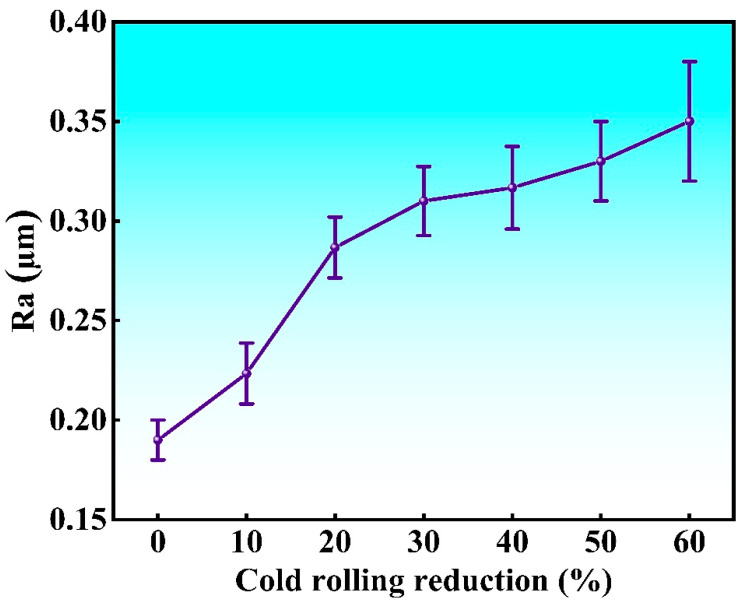
Surface roughness of the Invar alloy strips with different cold rolling reductions.

The 3D morphologies of the strips with different cold rolling reductions are characterized by LCM are shown in Figure 3; for each specimen a selected area measuring 120 µm × 120 µm was characterized. For the original strip, there are obvious parallel rolling scratches, while the surface undulation and height difference within the whole area are not so serious. After the cold rolling, many raised (red) and recessed (blue) regions appear, and the height difference increases sharply, which corresponding to the “pits” in Figure 2. Figure 4 shows the surface roughness of the strips measured by LCM, which reveals that the roughness increases monotonically from 0.18 µm to 0.35 µm with increasing cold rolling reduction, and the error bars also become larger as the different areas become less uniform in roughness. There are three reasons for the increase in roughness after cold rolling. Firstly, the non-uniform plastic deformation among different grains results in high internal stress and warping. Secondly, there are only a few grains within the thickness range of the strip, and thus, the elastic deformation at different regions can hardly be identical due to the different grain orientation, so different spring backs occur, and the thickness of the strip is not uniform, which is the primary cause of increased roughness. Thirdly, the formation of the high-density slip bands can also increase the surface roughness.

### 3.2. Evolutions of Grains and Intracrystalline Microstructure

**Figure 5:**
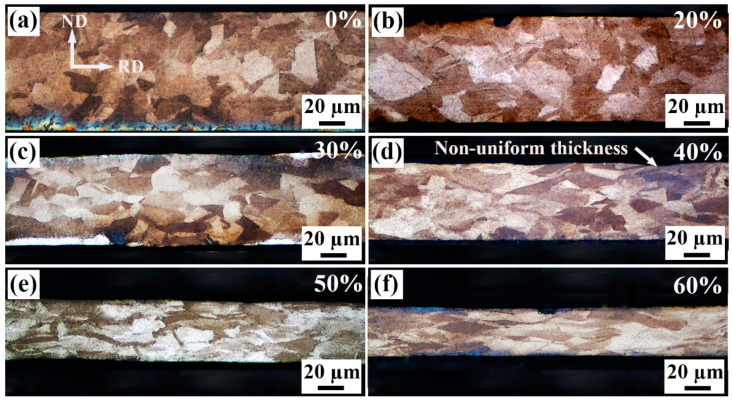
Cross-sectional morphologies of the Invar alloy strips with different cold rolling reductions (%): (**a**) original strip, 100 μm (0%), (**b**) 80 μm (20%), (**c**) 70 μm (30%), (**d**) 60 μm (40%), (**e**) 50 μm (50%) and (**f**) 40 μm (60%).

Figure 5 shows the cross-sectional images of the strips with different cold rolling reductions. For the original strip, the grains are equiaxed, and the thickness of the strip is uniform, whereas there are only four~six grains within the thickness range, and thus, the deformation resistance of different locations can hardly be uniform. With increasing cold rolling reduction, the grains are gradually compressed into elongated shape. Moreover, it is obvious that the thickness becomes non-uniform, as shown in Figure 5b–d, because the plastic deformation, rotation and spring back of the grains with different orientations are quite different. Some concavities or pits are formed on the surface of the strip, as marked in Figure 5d, which fit with the SEM images. Under tensile loading, cracking may occur firstly around these concavities due to stress concentration. With further increase in the cold rolling reduction, the grains become flatter, and the GBs are less clear (see Figure 5e,f).

**Figure 6:**
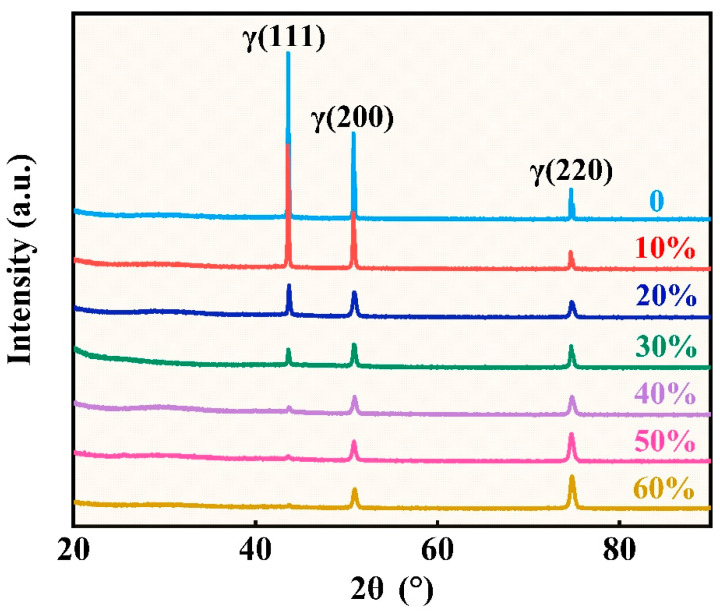
XRD patterns of the strips with different cold rolling reductions.

The XRD patterns of the strips with different cold rolling reductions are shown in Figure 6. It can be seen that the Invar alloy is a single phase, and the cold rolling will not cause phase transformation, while the relative intensities of the three diffraction peaks of the alloy change with increasing rolling reduction. For the original and 10% cold rolled strip, the relative intensity of the (111) diffraction peak is much higher, but it drops sharply when the rolling reduction increases to 20%. When the rolling reduction reaches 60%, the relative intensity of the (111) diffraction peak is almost negligible. The relative intensity of the (200) diffraction peak decreases firstly but becomes stable as the deformation further increases, while the relative intensity of the (220) diffraction peak shows a trend of decrease firstly and then increases, and its relative intensity is even higher than that in the original state when the deformation reaches 60%. The XRD patterns demonstrate the formation of texture during the cold rolling process.

**Figure 7:**
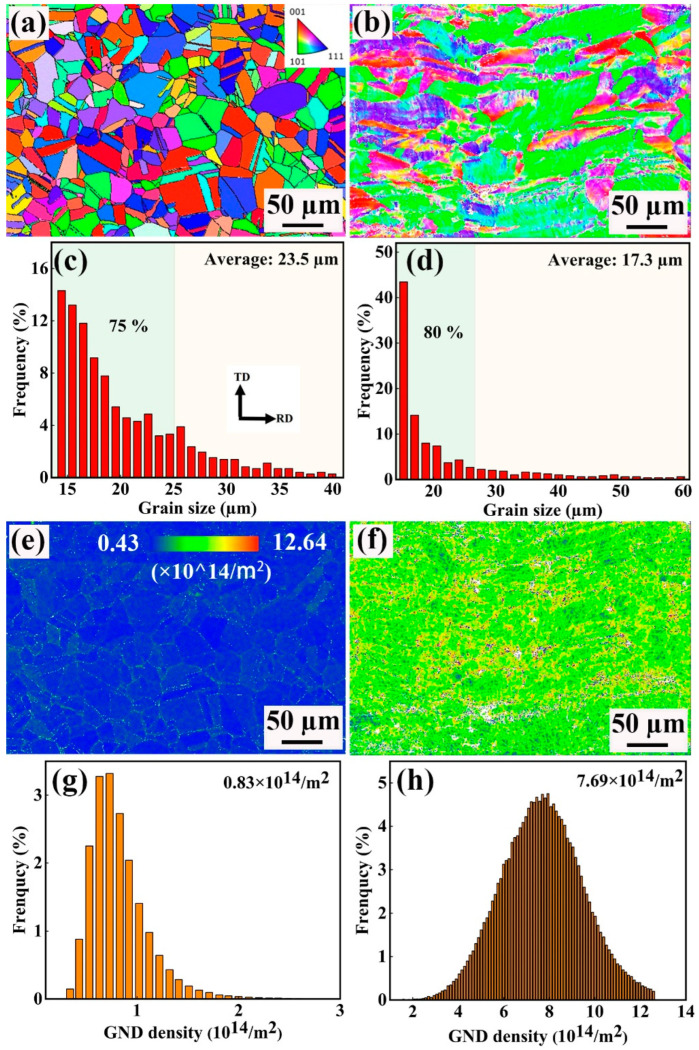
Grain and dislocation characteristics of the original and the 60% cold-rolled strips characterized by EBSD: (**a**,**b**) grain maps, (**c**,**d**) GB characteristics, (**e**,**f**) GND distribution maps, (**g**,**h**) GND statistical results.

The EBSD characterization results of the original and the 60% cold-rolled strips are shown in Figure 7. Figure 7a shows grain maps, from which it can be seen that the original strip is composed of equiaxed grains with randomly distributed orientation, with little annealing texture. After 60% of cold rolling reduction, texture is formed in the strip, which can be seen from the increase in the “green color”, i.e., the (110) orientation. As there is high inner stress in the cold-rolled strip, some areas cannot be identified by the EBSD, especially around the GBs; thus, the grain map in Figure 7b is not so clear. The distribution of grain size is shown in Figure 7c,d, which reveals that the average grain size decreases from 23.5 μm in the original strip to 17.3 μm in the 60% cold rolled strip, and the reason for this is that the fragmentation of some grains increases the total grain number. Meanwhile, some large grains are elongated, and their sizes increase. As a result, the difference in grain size becomes more obvious. The Geometrically Necessary Dislocations (GND) pattern is a technology used to analyze geometrically necessary dislocations in material [18,19]. Figure 7e,f shows the GND distribution maps and the statistical results of the original and the 60% cold-rolled strips, respectively, which reveal that the average dislocation density in the original strip is 0.83 × 10^14^/m^2^, and mainly exists around the GBs. In contrast, the average dislocation density in the 60% cold-rolled strip increases to 7.69 × 10^14^/m^2^, almost decuple that of the original strip. Additionally, it can be found that the dislocation density at different locations is not uniform and related to grain orientation.

**Figure 8:**
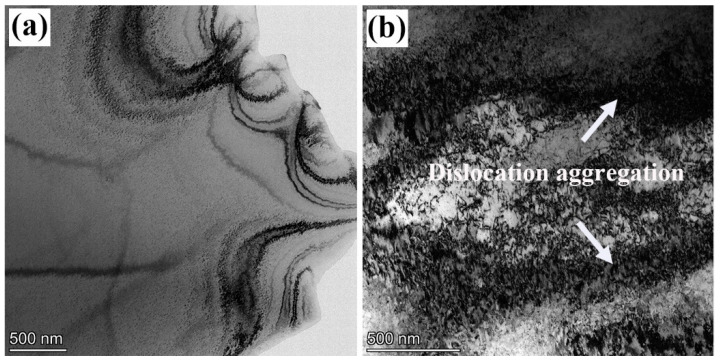
Intracrystalline microstructure of the grains in (**a**) the original strip and (**b**) the 60% cold-rolled strip.

Figure 8 shows the TEM images of the original and the 60% cold-rolled strips. For the original annealed strip, it is quite clear inside the grain, with few dislocations, as shown in Figure 8a. After 60% cold rolling, tangles of high-density dislocation were observed in the grain (see Figure 8b) because there is continuous dislocation multiplication but no recovery during the cold rolling. The increase in dislocation density promotes interactions between dislocations and forms complex structures inside the material [20], such as dislocation crossing, entanglement and intersection, which can effectively hinder further slips of dislocation and thereby enhance the alloy strength [21,22]. However, the dislocation interactions and pinning will surely decrease the plasticity and make the alloy more prone to local brittle fracture; thus, the mechanical properties of the strips were tested.

### 3.3. Mechanical Properties and Fracture Behavior

**Figure 9:**
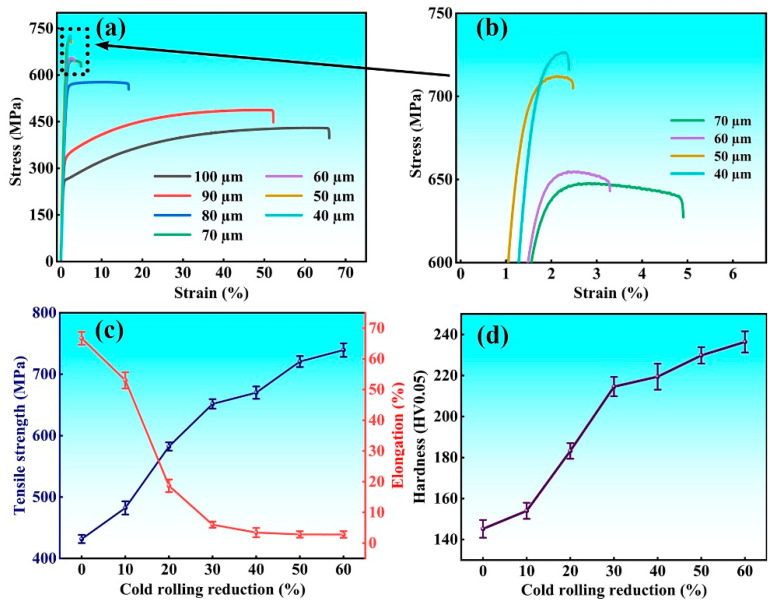
Mechanical properties of the Invar alloy strips after different cold rolling reductions: (**a**,**b**) tensile curves, (**c**) tensile strength and elongation, (**d**) microhardness.

The tensile curves, strength, elongation and microhardness of the Invar alloy strips after different cold rolling reductions are shown in Figure 9. It can be seen from the tensile curves that the tensile strength increases significantly, and the elongation decreases with increasing cold rolling reduction, while there are different stages. When the strip was rolled from 100 µm to 70 µm, the strength increased linearly from 430 MPa to 650 MPa, and the elongation decreased linearly from 65.4% to 8.5%. With further increase in the cold rolling reduction from 30% to 60%, the strength still increases but not so fast compared with the previous stage, and the elongation decreases to a very low level. For the 60% cold-rolled strip, a tensile strength of 738 MPa and an elongation rate of 2.8% were obtained. The evolution in microhardness of the strip is similar to that of tensile strength, as shown in Figure 9d.

**Figure 10:**
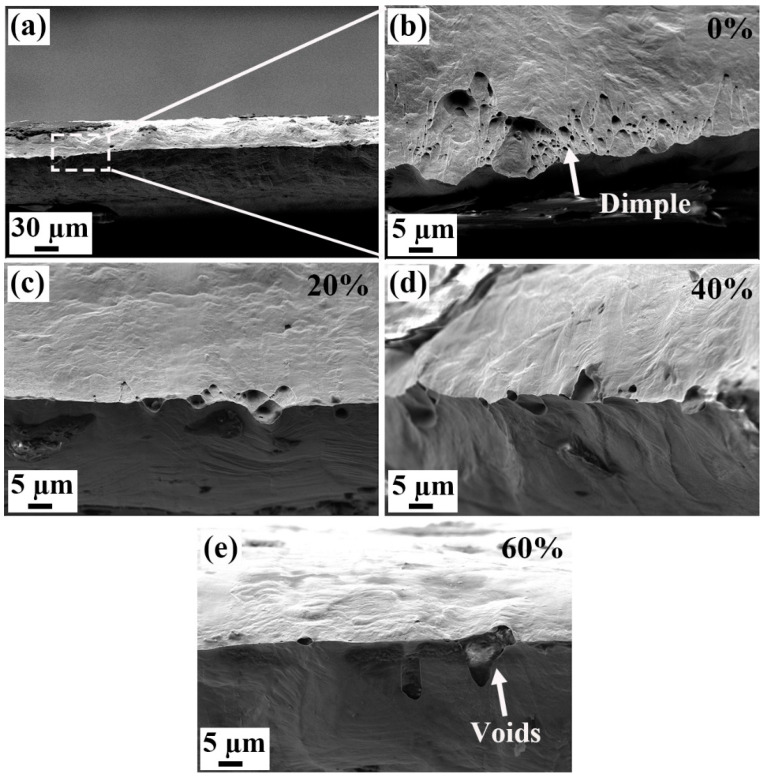
Tensile fracture surfaces of the Invar alloy strips with different cold rolling reductions (%): (**a**,**b**) original strip, 100 μm (0%), (**c**) 80 μm (20%), (**d**) 60 μm (40%) and (**e**) 40 μm (60%).

**Figure 11:**
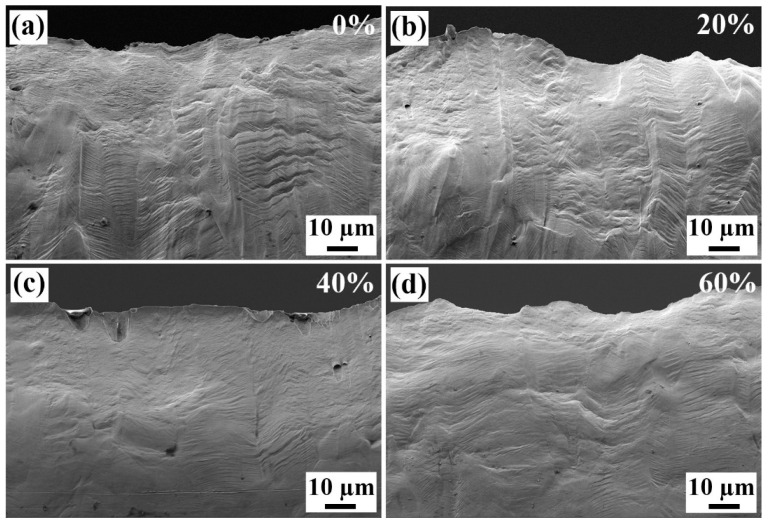
Tensile side surfaces of the Invar alloy strips with different cold rolling reductions (%): (**a**) original strip, 100 μm (0%), (**b**) 80 μm (20%), (**c**) 60 μm (40%) and (**d**) 40 μm (60%).

Figure 10 and Figure 11 show the tensile fracture surfaces and side surface morphologies of the strips. The original Invar alloy strip shows excellent plasticity and good ductility, and the necking is so serious that the fracture shows a “knife-edge” shape, as shown in Figure 10a. In microscopic, a large number of small-sized dimples exist on the fracture surface (see Figure 10b), which is a typical ductile fracture. As the cold rolling reduction increases, the fracture surfaces still show a “knife-edge” shape, but few dimples exist on the fracture surface, as shown in Figure 10c–e. Figure 11 shows the side surfaces of the strips with different cold rolling reductions. It is interesting to find that although the tensile elongations of these specimens are quite different, the deformation morphologies are similar, i.e., there is little difference in density of slip bands on the side surfaces. Therefore, it can be predicted that the total plastic deformation of the strip during the cold rolling and the tensile processes are close. The plastic deformation during the cold rolling process will consume the plastic deformation potential of the strip. As a result, the original strip has an elongation of 65.4%, and that of the 60% cold-rolled strip is only 2.8%, while their total plastic deformation amounts are close. In fact, when further cold rolling was conducted, cracking along the GBs occurred.

### 3.4. Discussion

**Figure 12:**
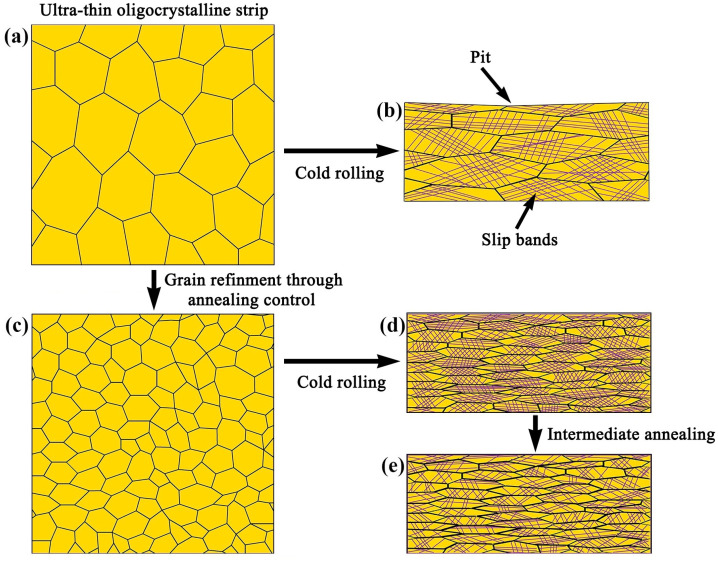
Illustration on the cold rolling deformation of the ultra-thin oligocrystalline Invar strip and strategies to improve the cold rolling deformation uniformity of ultra-thin oligocrystalline Invar alloy: (**a**) ultra-thin oligocrystalline strip, (**b**) oligocrystalline strip after cold rolling, (**c**) ultra-thin strip with finer grains, (**d**) fine-grained strip after cold rolling and (**e**) fine-grained strip after cold rolling and intermediate annealing.

The microstructure and property evolution of the ultra-thin oligocrystalline Invar36 alloys during the cold rolling process were comprehensively characterized, and their mechanical properties were tested. An illustration of the cold rolling deformation of the ultra-thin oligocrystalline Invar strip and strategies to improve the cold rolling deformation uniformity of ultra-thin oligocrystalline Invar alloy are shown in Figure 12. It was found that when cold rolling reduction is low (<20%), the plastic deformation is relatively uniform. When the rolling reduction is further increased, the influences of grain orientation on the yielding and plastic deformation emerges, making the slip bands on the grain surfaces obviously different. Since there are only a few grains within the thickness range, the inhomogeneity of grain deformation during the cold rolling leads to the inhomogeneity of spring back and localized concavity (see Figure 12a,b), which increases the surface roughness and stress concentration under external loading. As there is grain rotation and the formation of texture after certain cold rolling, the plastic deformation resistance of different grains should tend to be uniform. However, the strain hardening degree of these grains are different as the strain accumulation is different in the grains, and there is no stress relief annealing. The high-cold-rolling plastic deformation results in high-density dislocations and a significant increase in tensile strength, but a sharp decrease in elongation. It seems that the total plastic deformation during the cold rolling and tensile processes is a certain value because the plastic deformation potential is limited [23,24].

To solve the problem of uneven deformation, the following methods are suggested: Firstly, the growth in grain size during the previous recrystallization annealing process should be strictly controlled in order to ensure there are over 10 grains within the thickness range, as shown in Figure 12a,c. In fact, a tunnel furnace is needed as the annealing time window is very narrow for such a thin strip [25]. Secondly, the intermediate annealing with proper temperature can be used to promote the recovery of the alloy grains with a higher hardening degree, but this will have little influence on the grains with relatively low plastic deformation degrees or dislocation density, as illustrated in Figure 12a,c; thus, such a quick annealing can decrease the difference in hardness of different grains [26,27]. As there is a limit for the deformation strengthening of the single-phase Invar alloy, a precipitation strengthening through microalloying might be applicable.

## 4. Conclusions

In this study, the evolutions in microstructure and mechanical properties of an oligocrystalline Invar36 alloy strip, 100 µm thick, during the cold rolling were comprehensively investigated. The main conclusions are as follows:

(1) Plastic deformation at different locations of the strip during the cold rolling is quite non-uniform, depending on the orientation and deformation resistance of the grains in each area, forming different slip band appearances, uneven thickness and increase in surface roughness. Grain rotation and rolling texture develop with increasing cold rolling reduction.
(2) The grains are elongated during the cold rolling, and fragmentation occurs only in grains with severe plastic deformation. The dislocation density in the 60% cold-rolled strip is almost decuple that of the original strip, forming dislocations tangles, but this is not uniform in different grains.
(3) The tensile strength increases from 430 MPa of the annealed strip to 738 MPa of the 60% cold-rolled strip, but the elongation decreases sharply from 65.4% to 2.8%. The total plastic deformation of cold rolling and tensile processes has a certain limit. Grain refining and proper intermediate annealing are proposed to optimize the thickness uniformity, evenness and surface roughness.

## Figures and Tables

**Table 1:** Chemical composition of the Invar alloy.

Elements	Ni	Mn	Si	Cu	S	Fe
Content (wt%)	36.2	0.331	0.239	0.11	0.09	Bal

## Data Availability

The original contributions presented in this study are included in the article. Further inquiries can be directed to the corresponding author.
